# Molecular epidemiology and phylogenetics of camel anaplasmosis

**DOI:** 10.1371/journal.pone.0331833

**Published:** 2025-09-18

**Authors:** Farhan Ahmad Atif, Ammar Tahir, Muhammad Kashif, Aziz ur Rehman, Abdulmohsen H. Alqhtani, Alaa Bassuny Ismael, Ioannis A. Giantsis, Adil Khan, Furhan Iqbal, Muhammad Imran, Ayman A. Swelum

**Affiliations:** 1 Medicine Section, Department of Clinical Sciences, College of Veterinary and Animal Sciences, Jhang, Sub-Campus of University of Veterinary and Animal Sciences, Lahore, Pakistan; 2 Pathology Section, Department of Pathobiology, College of Veterinary and Animal Sciences, Jhang, Sub-Campus of University of Veterinary and Animal Sciences, Lahore, Pakistan; 3 Department of Animal Production, College of Food and Agriculture Sciences, King Saud University, Riyadh, Saudi Arabia; 4 Department of Clinical Laboratory Sciences, College of Applied Medical Sciences, Taif University, Taif, Saudi Arabia; 5 Department of Animal Science, Faculty of Agriculture, Forestry and Natural Environment. Aristotle University of Thessaloniki, Greece; 6 Department of Zoology, Bacha Khan University, Charsadda, Khyber Pakhtunkhwa, Pakistan; 7 Institute of Zoology, Bahauddin Zakariya University Multan, Multan, Pakistan; 8 Department of Parasitology, University of Agriculture, Faisalabad, Pakistan; National Research Centre, EGYPT

## Abstract

Camel anaplasmosis is a tick-borne disease of zoonotic concern, yet its epidemiology in Pakistan remains understudied. This study aimed to determine the prevalence, associated risk factors, and phylogenetic characteristics of Anaplasma spp. in camels across diverse agro-climatic zones of Punjab. A total of 400 blood samples were collected from two districts—Jhang and Bahawalpur (n = 200 each)—using a multistage cluster sampling approach. From each district, four tehsils were selected; ten herds per tehsil were sampled, with five camels per herd. The PCR targeting the *16S rRNA* gene was used for Anaplasma detection. Epidemiological data were gathered using a structured questionnaire. The overall prevalence was 25.75%. Multivariable analysis identified age (>5 years), district (Jhang), intensive management, and health status as significant risk factors. Phylogenetic analysis revealed that *A. phagocytophilum* isolates were genetically related to strains from India, Iran, and Turkey; *A. platys* showed proximity to dog-derived isolates from India, South Africa, and Spain; while *Candidatus A. camelii* was closely related to camel isolates from Egypt, China, Kenya, and Iran. In conclusion, camel anaplasmosis is prevalent in Punjab. Further research is warranted to explore the pathogenic potential and vector dynamics of circulating strains to devise control strategies.

## Introduction

Ticks are important vectors of various bacterial, protozoal, viral, and parasitic pathogens, posing serious health risks to both animals and humans [1]. Among tick-borne diseases, anaplasmosis—caused by *Anaplasma* species—is particularly significant among ruminants such as cattle, camels, sheep, and goats. This results in notable economic losses, especially in tropical and subtropical regions [2]. Pathogens like *Anaplasma*, *Ehrlichia*, *Rickettsia*, *Bartonella*, *Borrelia*, *Theileria*, and *Babesia* are transmitted between ticks and hosts, often leading to systemic illness [3].

*Anaplasma* spp., members of the family *Anaplasmataceae*, are gram-negative, obligate intracellular bacteria that predominantly infect camel blood cells, causing extravascular hemolysis and clinical signs such as fever, anorexia, jaundice, emaciation, anemia and weight loss [4,5]. Camel anaplasmosis mostly remains asymptomatic. Major species that can infect camels include *A. marginale*, *A. centrale*, *A. ovis*, *A. platys*, and *A. phagocytophilum* [6,7]. These pathogens generally circulate silently among tick and camelid hosts. The *A. phagocytophilum* is one of the most diverse pathogen, infecting a wide range of vertebrate hosts [4]. Anaplasmosis in camels can lead to reduced productivity, reproductive losses, and economic losses, especially for small-holder farmers in arid regions [8]. Transmission is influenced by numerous ecological and demographic factors, including camel movement, vector abundance, climate change, and land use [9–11]. Vectors such as *Rhipicephalus*, *Hyalomma*, *Ixodes*, *Dermacentor*, and *Argas* ticks are primarily responsible for the spread of tick-borne diseases namely anaplasmosis, babesiosis, theileriosis and ehrlichiosis [12–14]. Transmission can also occur via biting flies, transplacental routes, or iatrogenic means [15–17].

Camels, well adapted to arid and semi-arid regions, are vital to the socio-economic fabric of pastoral communities, providing transport, milk, and meat [18,19]. Pakistan hosts over 1.2 million camels, across 20 breeds, primarily in desert and semidesert zones [20,21]. Camel milk is highly nutritious. This animals’ unique physiological traits—such as efficient water conservation and thermoregulation—enable them to survive prolonged periods without food or water [19,22,23].

In Pakistan, available data on camel anaplasmosis are sparse and inconsistent, varying in study design, location, and diagnostic approaches [8,24]. Given the economic and epidemiological importance of this disease, the objective of the present study was to assess the prevalence, risk factors, and genetic diversity of *Anaplasma* spp. in camels from distinct agro-climatic zones of Punjab.

## Materials and methods

### Study area and sampling

**Fig 1:**
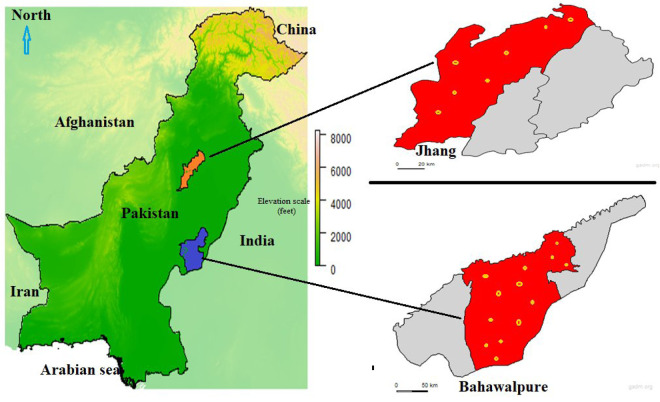
Maps of Pakistan showing the sampling districts: Jhang (highlighted in orange) and Bahawalpur (highlighted in blue). Enlarged district maps illustrate the specific sampling sites within Jhang and Bahawalpur..

This surveillance study was conducted in the Jhang and Bahawalpur districts of Punjab, Pakistan. These regions were characterized as arid and semiarid agro-ecological zones with high camel populations. Jhang (31.27°N, 72.33°E; 158 m a.s.l.) lies between the Jhelum and Chenab rivers and experiences a hot, humid climate from April to September, with milder temperatures during spring and autumn. Average summer temperatures range from 12.8–21.1°C (55–70°F), while winters are short, dry, and relatively cold (5.6–12.8°C or 42–55°F), with an annual rainfall of 22.5 mm (0.89 inches). Bahawalpur (117 m a.s.l.) has a dry climate with minimal rainfall (~143 mm/year) and an average annual temperature of 25.7°C (78.3°F). The raw maps were downloaded from https://en.wikipedia.org (Fig 1). Ethical clearance for this study was granted by the Directorate of Advanced Studies, University of Veterinary and Animal Sciences, Lahore, Pakistan, as documented in letter no. DAS/1468; dated August 24, 2023. All the methods were performed in accordance with ARRIVE guidelines laws and regulations. A total of 400 blood samples were collected from camels (n = 200 per district) using a multistage cluster sampling method. Four tehsils were randomly selected from each district. From each tehsil, 10 herds were chosen, and five camels were sampled per herd. Jugular blood was aseptically collected into EDTA-containing vacutainers and immediately stored in iceboxes for transport.

### DNA extraction and PCR

Genomic DNA was extracted from blood samples using the Gene JET Whole Blood Genomic DNA Purification Mini Kit (Thermo Fisher Scientific), following the manufacturer’s protocol. Extracted DNA was stored at –20°C until further use.

The *16S rRNA* gene was amplified using genus-specific primers: forward 5′-GGTACCYACAGAAGAAGTCC-3′ and reverse 5′-TAGCACTCATCGTTTACAGC-3′, as described by Azmat et al. [24]. The PCR reactions were performed at a total volume of 20µl. Each reaction mixture consisted of 10µl of TOPreal™ qPCR 2x PreMIX, 2µl of DNA template, 2µl (10 pmol) of each primer, and was brought to the final volume with 4µl of distilled water. The thermal cycling program began with an initial denaturation phase at 95°C for 5 minutes. Subsequently, 35 cycles were performed, each comprising an annealing step at 58°C for 30 seconds and an extension step at 72°C for 30 seconds. The protocol concluded with a final elongation step at 72°C for 10 minutes. Each PCR protocol has a positive and negative control (sterile nuclease free water). The PCR amplification products were separated in a 1.3% agarose gel stained with ethidium bromide (0.5 µg/µL) in a 1X TAE buffer, using a 100 bp DNA ladder as a size marker. Gel documentation was done using a trans-illuminator.

### Assessment of risk factors

A pre-validated, structured questionnaire was used to collect epidemiological data at the time of sampling. Risk factors included in the study were district (Jhang, Bahawalpur), feeding type (stall-fed, free-grazing), presence of other livestock, housing type (brick/wood vs. concrete/metal), tick burden (low, moderate, heavy), gender, acaricide use, age (<5 vs. > 5 years), management system (intensive, semi-intensive), presence of wall cracks, health status (healthy, emaciated), housing (covered vs. semi-covered), grooming practices, and dung disposal location (near to animals vs. far animals). Associations between these factors and Anaplasma infection were assessed using Chi-square analysis in IBM SPSS v26.

### Sequencing and phylogenetic analysis

Selected PCR-positive samples were sequenced by Macrogen Inc. (South Korea). Sequences obtained for *Candidatus A. camelii* (OR643816), *A. platys* (OR614083), and *A. phagocytophilum* (OR614030) were compared with homologous sequences using NCBI BLASTn. Similar sequences were retrieved from the NCBI nucleotide database for phylogenetic analysis.

Multiple sequence alignment was conducted using MUSCLE in MEGA 11 software under default settings. Phylogenetic trees were constructed using the Maximum Likelihood method based on the Tamura-Nei model [25]. The Tamura-Nei model is a method used to study how DNA sequences evolve. It’s more realistic than basic models because it recognizes that not all DNA mutations happen at the same rate. This helps to create more accurate evolutionary trees. The heuristic search was initiated using Maximum Parsimony and refined by Nearest-Neighbour Interchange. Node support was evaluated via 1000 bootstrap replicates [26].

### Statistical analysis

Chi-square tests were used to assess associations between categorical variables and disease prevalence. Both univariate and multivariate logistic regression analyses were conducted using SPSS v26 to identify significant risk factors. Variables with a p-value ≤ 0.05 and odds ratio (OR) >1 were considered statistically significant.

## Results

### Molecular prevalence

In this study, PCR targeting the *16S rRNA* gene detected Anaplasma spp. in 103 out of 400 camel blood samples, yielding an overall prevalence of 25.75%. The PCR-based prevalence of camel anaplasmosis in Jhang and Bahawalpur was 21% (42/200) and 30.5% (61/200), respectively. A significantly higher prevalence was observed in Bahawalpur compared to Jhang (p = 0.039, χ² = 4.72, df = 1).

Camels kept with other livestock showed a significantly higher infection rate (32.72%) than those reared alone (17.22%) (p = 0.001, χ² = 12.44, df = 1). Housing type was also associated with infection in camels in woody-brick structures. This had a higher prevalence (31.30%) than those in concrete-metallic shelters (18.23%) (p = 0.004, χ² = 8.73, df = 1).

Tick burden showed a graded association: camels with heavy, moderate, and low infestations had infection rates of 34.93%, 29.16%, and 19.79%, respectively (p = 0.01, χ² = 8.05, df = 2). Camels not treated with acaricides had a significantly higher prevalence (31.37%) compared to those that received acaricide treatments (19.27%) (p = 0.006, χ² = 8.10, df = 1).

Older camels (>5 years) were more frequently infected (19.65%) than younger ones (17.18%) (p = 0.01, χ² = 5.90, df = 1). Infection was notably higher in camels under semi-intensive management (44.02%) compared to those under intensive care (13.69%) (p < 0.001, χ² = 46.09, df = 1). Similarly, emaciated camels had significantly higher infection rates (37.32%) than healthy ones (12.02%) (p < 0.001, χ² = 33.25, df = 1).

Grooming was also associated with the disease prevalence. The camels that underwent regular grooming had lower infection (20.23%) compared to ungroomed ones (29.95%) (p = 0.02, χ² = 4.58, df = 1). Other factors, including feeding type, gender, wall cracks, dung location, and housing area, showed no significant association with the occurrence of disease (Table 1).

**Table 1:** Chi square based analysis of determinants of camel anaplasmosis in Punjab, Pakistan.

Variables	Categories	Total no. of animals tested	No. of positive animals	Prevalence (%)	*X* ^2^	*p*- value	df
Types of feeding	Outdoor browsing	295	83	28.13	3.34	0.70	1
	Indoor feeding	105	20	19.04			
Other livestock with camels	Present	220	72	32.72	12.44	0.001	1
	Absent	180	31	17.22			
Housing type	Woody + bricks	230	72	31.30	8.73	0.004	1
	Concrete + metallic	170	31	18.23			
Tick infestation	Heavy	83	29	34.93	8.05	0.01	2
	Moderate	120	35	29.16			
	Low	197	39	19.79			
Gender	Male	140	43	30.71	2.77	0.11	1
	Female	260	60	23.07			
Use of acaricides	Yes	192	37	19.27	8.10	0.006	1
	No	208	66	31.37			
Age	> 5 years	173	34	19.65	5.90	0.01	1
	< 5 years	227	39	17.18			
Area	Jhang	200	42	21	4.72	0.039	1
	Bahawalpur	200	61	30.5			
Management types	Intensive	241	33	13.69	46.09	0.00	1
	Semi-intensive	159	70	44.02			
Cracks in the walls	Yes	273	77	28.20	2.71	0.11	1
	No	127	26	20.47			
Health status	Healthy	183	22	12.02	33.25	0.00	1
	Emaciated	217	81	37.32			
Animal living area	Open	283	59	20.84	0.28	0.64	1
	Congested	162	44	27.16			
Grooming practice	Yes	173	35	20.23	4.58	0.02	1
	No	227	68	29.95			
Dung location	Near to animals	197	58	29.44	2.76	0.11	1
	Far from animals	203	45	22.16			

### Risk factor analysis

Univariate logistic regression identified several significant predictors of anaplasmosis in camels: acaricide use (p = 0.051, OR=1.788, 95% CI: 0.997–3.207), age > 5 years (p = 0.009, OR=2.196, CI: 1.212–3.979), district (Jhang) (p = 0.001, OR=2.695, CI: 1.475–4.925), semi-intensive management (p < 0.001, OR=5.629, CI: 3.093–10.244), poor health (p < 0.001, OR=4.850, CI: 2.472–3.915), and lack of grooming (p = 0.001, OR=2.846, CI: 1.528–5.303) (Table 2).

**Table 2:** Univariate and multivariate logistic regression analysis of risk factors related to anaplasmosis in camels.

Univariate logistic regression						
**Variable**		**B**	***p*-value**	**OR**	**Lower CI**	**Upper CI**
Types of feeding		−0.85	0.02	0.427	0.208	0.876
Other livestock with camels		−1.394	0.000	0.248	0.135	0.457
Housing type		−0.527	0.083	0.591	0.326	1.071
Tick infestation		−0.379	0.035	0.685	0.481	0.973
Gender		−0.007	0.983	0.993	0.547	1.803
Use of acaricides		0.581	0.051	1.788	0.997	3.207
Age		0.787	0.009	2.196	1.212	3.979
Area		0.992	0.001	2.695	1.475	4.925
Management type		1.728	0.000	5.629	3.093	10.244
Cracks in the wall		−0.745	0.021	0.475	0.252	0.895
Health status		1.579	0.000	4.85	2.472	9.515
Animal living area		−0.205	0.518	0.815	0.437	1.517
Grooming practice		1.046	0.001	2.846	1.528	5.303
Dung location		−0.401	0.179	0.669	0.373	1.202
**Multivariate logistic regression**						
**Variable**	**Category**	**B**	***p-*value**	**OR**	**Lower CI**	**Upper CI**
Age	> 5 years	0.87	0.002	2.387	1.371	4.157
	< 5 years	—	—	—	—	—
Area	Jhang	0.918	0.001	2.504	1.449	4.328
	Bahawalpur	—	—	—	—	—
Management types	Intensive	1.523	0.000	4.588	2.682	7.848
	Semi-intensive	—	—	—	—	—
Health status	Healthy	1.618	0.000	5.041	2.752	9.232
	Emaciated	—	—	—	—	—

B = regression coefficient, OR = Odds Ratio, CI = Confidence Interval (95%).

Multivariate analysis confirmed four significant predictors associated with the occurrence of anaplasmosis in camels. Camels older than five years were at significantly higher risk (p = 0.002, OR=2.387, 95% CI: 1.371–4.175). The likelihood of infection was also higher in camels from the Jhang district (p = 0.001, OR=2.504, CI: 1.449–4.328). Management type showed a strong association, with camels reared under semi-intensive systems being more vulnerable to infection (p < 0.001, OR=4.588, CI: 2.682–7.848). Additionally, poor health status emerged as a significant risk factor, with emaciated camels being more frequently infected (p < 0.001, OR=5.041, CI: 2.752–9.232) (Table 2).

### Sequencing and phylogenetic analysis

**Fig 2:**
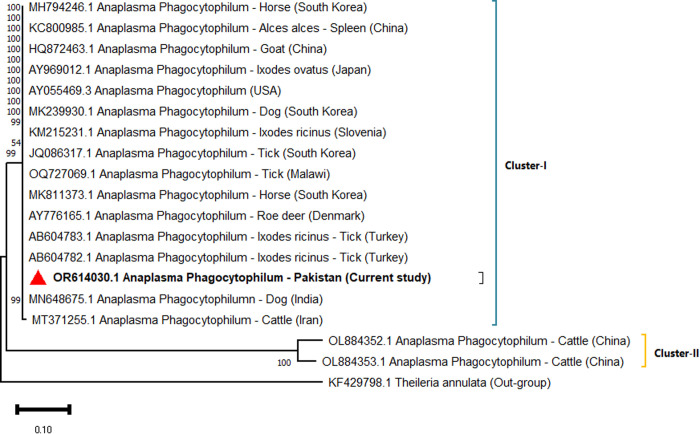
Maximum likelihood phylogenetic tree of *A. phagocytophilum* based on the *16S rRNA* gene.

**Fig 3:**
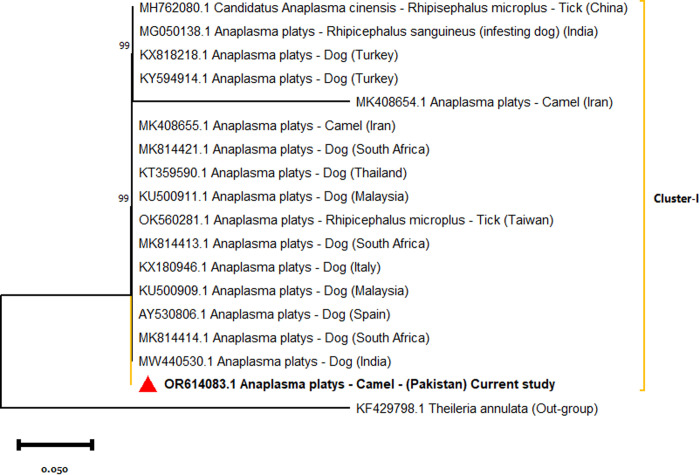
Maximum likelihood phylogenetic tree of *A. platys* based on the *16S rRNA* gene.

**Fig 4:**
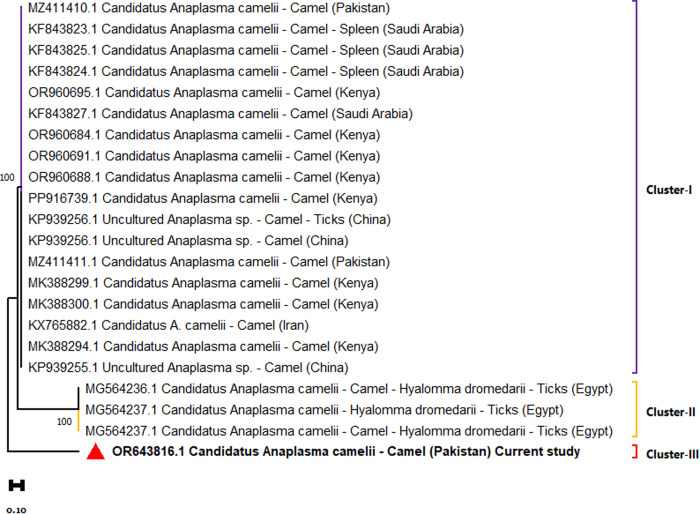
Maximum likelihood phylogenetic tree of Candidatus *A. camelii* based on the *16S rRNA* gene.

The PCR-positive samples were confirmed through sequencing, and three representative isolates were submitted to GenBank: *Anaplasma phagocytophilum* (OR614030), *Anaplasma platys* (OR614083), and *Candidatus Anaplasma camelii* (OR643816). Phylogenetic trees were constructed using the Maximum Likelihood method based on the Tamura–Nei model. The *A. phagocytophilum* isolate from Pakistan clustered closely with strains from India, Iran, and Turkey (Cluster I) with bootstrap value of 99 with the main clade, while it remained genetically distinct from Chinese cattle-derived isolates (Cluster II) with bootstrap value of 100, forming a single major clade (Fig 2). The *A. platys* sequence was grouped with isolates from India, South Africa, Turkey, Iran, Malaysia, and Spain, most of which were obtained from dogs and ticks with bootstrap values ranging from 99–100 (Fig 3). This tree formed a single major clade. For *Candidatus A. camelii*, three distinct clusters were identified. Pakistani isolates (including OR643816) are grouped within single clade forming two clusters (Cluster I and Cluster II). The Cluster I (bootstrap value 100), alongside sequences from Saudi Arabia, Kenya, China, and Iran, are primarily derived from camel blood and ectoparasites. Egyptian isolates obtained from *Hyalomma dromedarii* ticks formed a separate cluster (Cluster II, bootstrap values range from 99–100) (Fig 4). This study confirmed the presence of *A. phagocytophilum*, *A. platys* and *Candidatus A. camelii* in camels from these districts of Punjab, Pakistan.

## Discussion

Tick-borne diseases represent a major challenge to global livestock health and productivity. Veterinary public health efforts primarily aim to enhance animal well-being and reduce economic losses through targeted interventions. Camels, which are vital for milk, meat, and draught purposes, are increasingly affected by tick-borne pathogens that compromise their health and performance [18].

Anaplasmosis is a zoonotic tick-borne disease caused by several *Anaplasma* species, notably *A. centrale*, *A. marginale*, *A. phagocytophilum*, and *A. platys*, which can infect camels [27]. *A. phagocytophilum* and *A. platys* have zoonotic implications. Nevertheless, *A. phagocytophilum* is one of the most diverse pathogen, infecting a wide range of domestic and wild vertebrate hosts, including sheep, cattle goat, horse, donkey, dogs, cats, and wild ruminants as well as humans. In humans, it causes human granulocytic anaplasmosis with the main clinical symptoms of fever, chills, headache, and myalgia [4,5]. In Pakistan *A. phagocytophilum* has been detected in horses (10.67%), and domestic cats (7.03%) [28,29] as well as in camels in the current study. Age, previous tick history, sex, tick infestation, housing type, cracks in walls, rearing system, and tick control status were the major risk factors associated with anaplasmosis in equines. Whereas housing type, previous tick history, acaricide use, and grooming practices were the significant risk factors in cats [28,29]. The *A. platys* mainly infects dogs (canids) and camels. Human infections with *A. platys* are less common. Clinical signs in humans include fever, headache and myalgia [30]. Increasing infections in camels can be a public health concern. Identifying risk factors is critical for understanding disease dynamics and guiding control strategies [31].

In the present study, the molecular prevalence of camel anaplasmosis was found to be 25.75% notably higher than the 13.33% prevalence previously reported in a cross sectional survey from small holder farmers using *16S rRNA* based PCR from Mianwali district of northwestern Punjab, Pakistan [24]. The reason for lower disease occurrence in Mianwali was likely due to the fact that they took small sample size from small holder draft camels. Because small holders adopt better feeding and management practices compared to large herd size. Additionally, younger age, lower tick infestation and indoor feeding and better housing by the camel farmers of Mianwali region possibly contributed to the lower disease outcome. Though other reports depicted camel anaplasmosis ranging from 11.8% to 42.39% using various serological and molecular diagnostic approaches during transversal surveys [32–39].

This is the first molecular investigation focusing on camel anaplasmosis in two agro-ecologically distinct regions of Punjab, located around the Thal and Cholistan deserts, where camel populations are dense. Several biotic and abiotic risk factors were examined, including feeding type, herd composition, housing structure, tick burden, acaricide use, age, location, management style, wall integrity, animal condition, sheltering, grooming, and dung disposal. The study found higher disease prevalence in Bahawalpur (30.5%) compared to Jhang (21%). This could be attributed to variation in climatic conditions, higher camel population, and increased camel movement, especially near border regions where camels are used extensively by border security forces. A recent study depicted higher infection rate 42.72% in dromedary camels in India [40]. Tick abundance due to environmental conditions may also explain these findings with higher tick infestation rate in Bahawalpur (74.81%) compared to Jhang (26%) [41,42].

Significant associations were observed between anaplasmosis and co-rearing with other livestock, housing type, tick load, acaricide use, age, location, management system, body condition, and grooming practices. Although camels fed through outdoor browsing showed higher infection rates (28.13%), this difference was not statistically significant. Similar trends were reported by Azmat et al. [8] and Noaman [43], while contrasting reports suggest outdoor-fed camels may be less affected [44,45], possibly due to differing exposure levels. Co-rearing camels with other livestock had increased infection risk, likely due to increased tick vector diversity and interspecies transmission. Camels housed in wood-brick structures with wall cracks were more susceptible, possibly due to favorable tick refuges that limit the effectiveness of control measures.

A positive correlation between tick infestation and disease occurrence was observed, aligning with earlier reports [4]. Most camels examined were infested with hard ticks, known vectors of anaplasmosis. Males were more frequently infected than females, possibly due to their greater use in transportation and mobility, which increases exposure. This is consistent with findings by Javed et al. [46] and Azmat et al. [8], although other studies [38,47] reported higher prevalence in females, potentially linked to immunosuppression during pregnancy.

Camels not treated with acaricides had significantly higher infection rates, reinforcing findings by Selim et al. [48] and Aslam et al. [49], who emphasized the effectiveness of acaricide use in controlling tick-borne diseases. Contrary to some reports suggesting higher infection in young animals due to weaker immunity [38,50], this study found older camels (>5 years) were more affected, possibly due to prolonged exposure and small sample size of younger camels.

Management practices also influenced disease dynamics. Semi-intensively managed camels had significantly higher infection rates, likely due to greater environmental exposure. Emaciated camels showed higher disease prevalence, highlighting poor health status as a key risk factor. Similar findings were reported by Azmat et al. [8] and Onyiche et al. [35]. Groomed animals had a lower infection rate, possibly because grooming helps in early tick removal and reduces disease transmission, as noted in previous studies [8,38].

Phylogenetic analysis revealed that *A. phagocytophilum* isolates from Pakistan were closely related to Indian, Iranian, and Turkish strains. The Chinese isolates showed higher genetic diversity compared to their counterparts from other countries. The current *A. phagocytophilum* isolates showed 100% homology with majority of the global isolates. The *A. platys* isolates clustered with sequences from India, South Africa, and Spain, mainly derived from dogs with 100% identity. Likewise, camel *A. platys* isolates from Iran showed significant heterogeneity compared to other group members. *Candidatus A. camelii* isolates were genetically similar to those from Egypt, China, Kenya, and Iran, sourced from dromedary camels and *Hyalomma dromedarii* ticks with 97.15% genetic similarity. Nonetheless, Egyptian isolates expressed higher divergence compared to other *Candidatus A. camelii* isolates as well as higher genetic diversity compared to other Anaplasma isolates detected in the present study. There are indications of genetic recombination; the implications of this can lead to higher genetic diversity and clinical complications [14,51].

## Conclusions

The findings confirm that camel anaplasmosis is prevalent in Punjab. Key risk factors include location (Jhang), age over five years, semi-intensive management systems, and poor health status. Phylogenetic insights revealed regional genetic links of *A. phagocytophilum*, *A. platys*, and *Candidatus A. camelii* with strains from neighboring and distant countries. Further research is needed to assess the clinical impacts and vector competence of these isolates to guide control strategies and treatment protocols.

## Supporting information

File 1 Inclusivity in global research. (DOCX)
